# The Study of Tropical Medicine

**Published:** 1915-09-04

**Authors:** 


### THE STUDY OF TROPICAL MEDICINE.

### Some Colonial Office Requirements.

At the London School of Tropical Medicine the sessions, three a year, extend over three months each. The Colonial Office requires that men selected for posts in the tropics must have attended one such session, and, in addition to showing regularity in attendance, must have passed the examination at the end of the course. Failure to do so involves loss of the appointment. Other students may take the examination or not, as they choose, and, if successful, they are entitled to a certificate to that effect. The courses are so arranged as to equip men for the Diploma in Tropical Medicine and Hygiene granted by the University of Cambridge, the Diploma in Diseases and Hygiene of the Tropics granted by the Conjoint Board, and the M.D. of the University of London (Part VI.), Tropical Medicine. The student is required to attend from 10 to 4 or 5 daily during the three months. In the wards of the hospital that adjoins the school are always to be found a number of tropical cases newly arrived. The main laboratory accommodates sixty students; the old laboratories have been sub-divided for special purposes. There is resident accommodation for twenty students. Each resident student is provided with a bed-sitting room, and has the use of the common-rooms, such as the dining-room, smoking-room, library, etc., and access to the laboratories at all times. Women graduates are received as students. Last year 178 students passed through the school.

